# A pseudovirus-based method to dynamically mimic SARS-CoV-2-associated cell-to-cell fusion and transmission: A method to mimic SARS-CoV-2 cell-to-cell transmission

**DOI:** 10.3724/abbs.2023129

**Published:** 2023-07-06

**Authors:** Xiangpeng Sheng, Yi Yang, Fang Zhu, Fan Yang, Honghua Wang, Ronggui Hu

**Affiliations:** 1 Key Laboratory of Systems Health Science of Zhejiang Province School of Life Science Hangzhou Institute for Advanced Study University of Chinese Academy of Sciences Hangzhou 310024 China; 2 State Key Laboratory of Molecular Biology Shanghai Institute of Biochemistry and Cell Biology Center for Excellence in Molecular Cell Science University of Chinese Academy of Sciences Chinese Academy of Sciences Shanghai 200031 China; 3 State Key Laboratory of Animal Disease Control Harbin Veterinary Research Institute Chinese Academy of Agricultural Sciences Harbin 150069 China; 4 Department of Thoracic Surgery Ruijin Hospital Shanghai Jiaotong University School of Medicine Shanghai 200025 China; 5 School of Medicine Guizhou University Guiyang 550025 China

Severe acute respiratory syndrome coronavirus 2 (SARS-CoV-2), responsible for the COVID-19 pandemic, has caused tremendous global loss and continues to evolve to generate variants. Entry of SARS-CoV-2 into target host cells is primarily mediated by spike (S), which binds to the host receptor hACE2 and initiates virus-cell membrane fusion [1]. Most COVID-19 patients show pneumocyte syncytia in the lungs [2]. Cell fusion contributes to viral entry, cell-to-cell transmission and tissue damage, and thus attracts much attention. Because authentic SARS-CoV-2 live virions can only be handled in biosafety level-3 (BSL-3) facilities, many researchers have developed different assays to study cell fusion in BSL-1/2 by directly expressing S and hACE2 on mammalian cells [ 2‒ 5] . Briefly, in regular cell fusion assays, S-expressing cells and hACE2-positive cells are cocultured at approximately a 1:1 ratio, which induces cell-cell fusion and usually activates a fusion reporter. Although these strategies are useful, they cannot efficiently simulate cell-cell fusion and transmission in SARS-CoV-2 infection, in which virions from one target cell are transmitted to neighboring cells, resulting in syncytia. Here, we design a pseudovirus-based method to dynamically and highly mimic cell-to-cell fusion and virus transmission of SARS-CoV-2.

First, we generated spike-pseudotyped virions (S pseudovirions) in HEK293FT cells by co-transfecting three plasmids, including psPAX2, pCDH-sfGFP, and a plasmid expressing SARS-CoV -2 S into cells ( Figure 1A), and collected the viral supernatant, which is similar to a previous report [6]. S pseudovirus was found to efficiently infect hACE2-positive 293T cells (293T-hACE2) but not the control 293T or 293FT cell lines ( Supplementary Figure S1A,B), suggesting that S indeed envelops the pseudovirions. However, fluorescence microscopy revealed that the infection of 293T-hACE2 cells by pseudovirus supernatant could not trigger cell-cell membrane fusion events ( Supplementary Figure S1C). Given that the authentic SARS-CoV-2-infected host cells can continue to generate live virions to promote the formation of syncytia, we hypothesized that a cell producing pseudovirions may show a similar capacity to induce cell-cell fusion.

**Figure 1:**
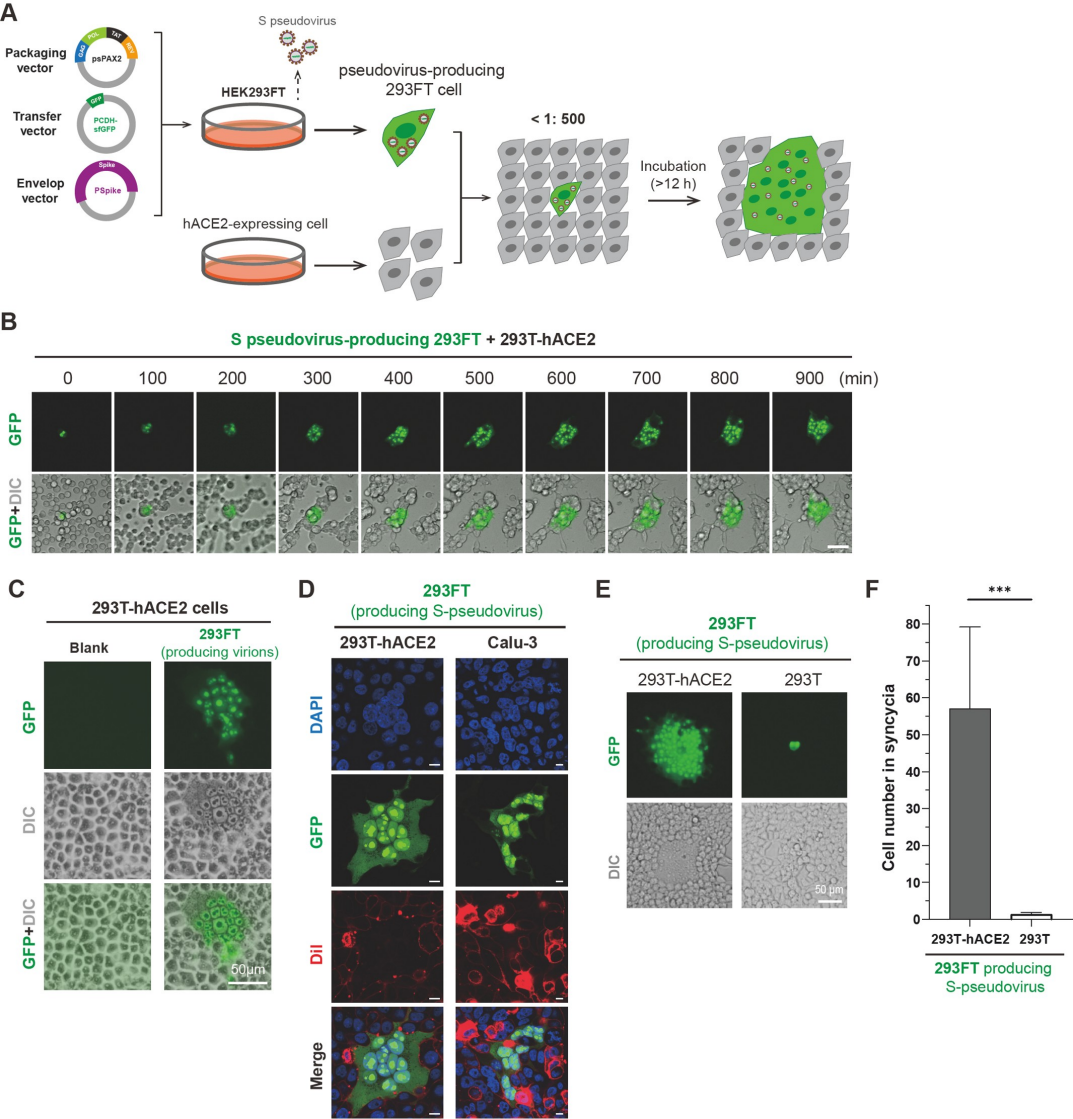
Figure 1 A novel strategy to mimic cell-cell fusion and transmission of SARS-CoV-2 (A) Schematic illustration of the pseudovirus-based cell-cell fusion assay. In brief, psPAX2, pCDH-sfGFP and an S-expressing plasmid were cotransfected into HEK293FT cells for 24 h. Pseudovirus-producing 293FT and hACE2-expressing 293T cells were cocultured at a ratio of approximately 1:500. Syncytia were monitored under a real-time imaging system or imaged at the indicated time points. (B) Real-time imaging of syncytium formation. Pseudovirus-producing 293FT and 293T-hACE2 cells were mixed and seeded in 29-mm dishes and monitored by the Olympus SpinSR real-time live cell imaging system. Scale bar: 50 μm. (C) The syncytia had a large cytoplasm containing multiple nuclei. The images were taken after 15 h of coculture of pseudovirus-producing 293FT and 293T-hACE2 cells under an Olympus IX73 microscope. Scale bar: 50 μm. (D) Confocal imaging of syncytia with nuclear and plasma membrane staining. Syncytia of 293T-hACE2 (left) or Calu-3 (right) cells were fixed with 4% PFA and then subjected to membrane staining (10 μM Dil, 30 min) and nuclear staining (DAPI). Scale bar: 10 μm. (E,F) Pseudovirus-producing 293FT cells cocultured with hACE2-negative 293T cells cannot induce cell fusion. Virion-producing 293FT cells were mixed with 293T or 293T-hACE2 for 24 h. (E) Microscopic images and (F) quantitative analysis of the relative sizes of syncytia. Scale bar: 50 μm. Data are presented as the mean±SD, *** P<0.001 (Student’s t test, n>10).

To test this hypothesis, pseudovirus-producing 293FT cells (GFP-positive) were digested into single cells after 24 h of transfection and were subsequently cocultured with 293T-hACE2 cells at a 1:500 ratio ( Figure 1A). The ratio of 293T-hACE2 cells to pseudovirus-producing 293FT cells in the mixture should be very high (approximately 500:1); otherwise, the formed syncytia may not be derived from a single pseudovirus-producing 293FT [5]. Real-time live cell imaging revealed that a single 293FT cell producing pseudovirions can readily induce large syncytium formation in 293T-hACE2 cells ( Figure 1B and Supplementary Video S1). The virion-producing GFP-positive 293FT cells can fuse with the closest neighboring cells first, which facilitates transmission of the pseudovirions to the neighboring cells and further promotes more and more cell-cell fusion. Moreover, the size of the GFP-positive syncytia was dependent on the coculture time ( Figure 1B). In the syncytia, multiple and separate nuclei were clearly observed to pack together ( Figure 1C). Confocal imaging of cells with nuclear and membrane staining further confirmed the membrane fusion of the syncytia ( Figure 1D). In addition, pseudovirus-producing 293FT cells also triggered syncytium formation in the Calu-3 cell line, a human bronchial epithelial cell line that endogenously expresses hACE2 ( Figure 1D), which suggests that any hACE2-expressing cells cocultured with virion-producing cells could form syncytia. Notably, unlike hACE2-expressing 293T cells, regular 293T cells cocultured with pseudovirus-producing 293FT cells did not exhibit syncytium formation ( Figure 1E,F), confirming that the syncytia were S/hACE2-driven cell-cell fusion. The data also excluded the possibility that the division or proliferation of virion-producing cells might contribute to syncytium formation. Therefore, a single cell producing S-pseudotyped virions can induce syncytia of hACE2-positive cells, which highly mimic authentic SARS-CoV-2-induced cell-cell fusion and transmission.

In a previous study, several of the most effective drugs, including niclosamide and salinomycin, were identified to inhibit syncytium expansion; the noncompetitive SERCA inhibitors thapsigargin (TG) and cyclopiazonic acid (CPA), which cause Ca ^2+^ depletion, also show an inhibitory effect on syncytium formation [2]. Our pseudovirus-based method was therefore utilized to test these potential drugs. Consistently, niclosamide, salinomycin and TG dramatically blocked the formation of syncytia mediated by wild-type (WT) S-pseudovirus-producing 293FT cells ( Figure 2A,B). CPA treatment also disrupted virion-mediated cell fusion, but its inhibitory function was much weaker than that of other drugs. Furthermore, this phenotype was recapitulated by Delta S-pseudovirus-producing cells ( Supplementary Figure S1D,E). Therefore, the pseudovirus-based strategy can be used to estimate the inhibitory effects of potential compounds that may restrict cell fusion, which makes it a powerful tool for drug screening.

**Figure 2:**
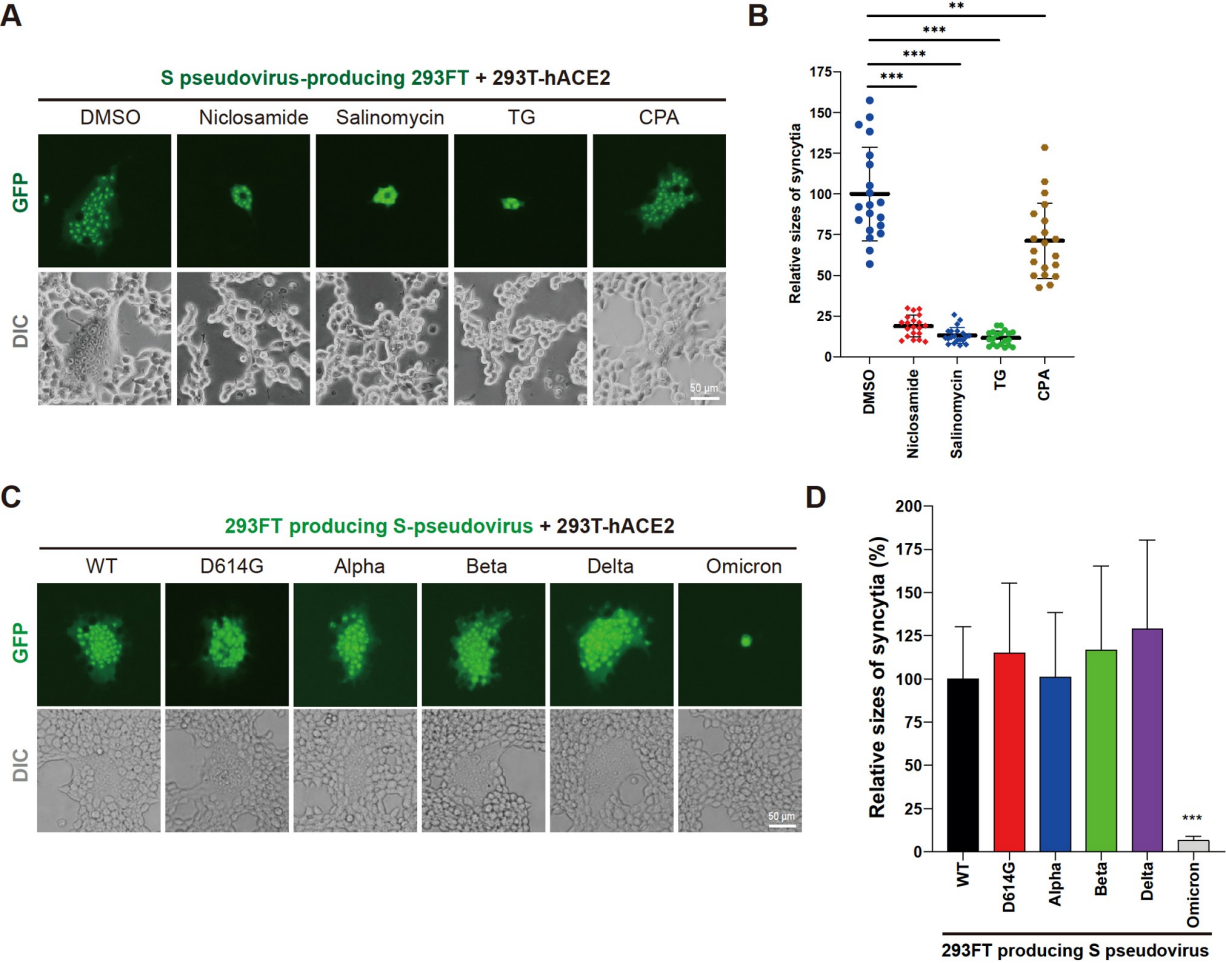
Figure 2 This pseudovirus-based strategy is a powerful tool for drug screening and SARS-CoV-2 investigation (A) This pseudovirus-based method is a powerful tool to estimate the inhibitory effects of potential drugs on S-mediated cell-cell fusion. DMSO, niclosamide (1 μM), salinomycin (1 μM), thapsigargin (TG; 1 μM) or cyclopiazonic acid (CPA; 5 μM) was added to the coculture system of pseudovirus-producing 293FT and 293T-hACE2 cells. Scale bar: 50 μm. (B) Quantitative analysis of the syncytia sizes in (A). Data are shown as the mean±SD, ** P<0.01, *** P<0.001 (Student’s t test, n=20). (C) This pseudovirus-based strategy revealed that Omicron had less fusogenic activity than early-pandemic SARS-CoV-2 variants. 293FT cells producing different S pseudovirions were separately cocultured with 293T-hACE2 for 24 h, and syncytia were imaged under an Olympus IX73 microscope. Scale bar: 50 μm. (D) Quantitative analysis of the syncytia sizes in (C). Data are presented as the mean±SD, *** P<0.001 (Student’s t test, n=10).

SARS-CoV-2 evolves to generate many variants of concern (VOCs) with increased infectious abilities and altered pathogenicity [7]. In particular, Omicron has been reported to be less fusogenic than early-pandemic SARS-CoV-2 variants [ 8, 9] . We constructed 293FT cells generating different pseudovirions carrying distinct S variants and conducted a cell-cell fusion assay. The results showed that while most S variants can induce significant syncytium formation, Omicron had a very weak ability to mediate cell-cell fusion ( Figure 2C,D), consistent with previous reports. Thus, this method shows the potential to explore the fusogenic capacity of SARS-CoV-2 variants.

Taken together, we developed a pseudovirus-based method to dynamically investigate cell fusion and cell-to-cell transmission of SARS-CoV-2, which will be useful for researchers who do not have access to a BSL-3 facility. This method can be used for high-throughput screening of drugs that have the ability to inhibit S mutant-induced syncytium formation and virus transmission. However, to provide sufficient area for the formation of at least 3 syncytia in each well, 48- or 96-well plates, but not 384-well plates, should be used in a high-throughput screening assay, which may increase reagent costs and screening time; moreover, it may be difficult to ensure the same syncytia numbers in each well. Importantly, given that other coronaviruses may have similar features to result in cell-cell fusion and syncytium formation in the host, we believe that the strategy we designed here will be very beneficial to scientific research on SARS-CoV-2 variants or future emerging coronaviruses.

## Supporting information

23276supplementaryVedio_S1

23276Supplementary_materials

## Supplementary Data

Supplementary data is available at *Acta Biochimica et Biophysica Sinica* online.
