# Supplementary material for: A pseudovirus-based method to dynamically mimic SARS-CoV-2-associated cell-to-cell fusion and transmission: A method to mimic SARS-CoV-2 cell-to-cell transmission
Source: Acta Biochim Biophys Sin (Shanghai). 2023 Jul 6;55(11):1840–3. doi: 10.3724/abbs.2023129 (PMC10679870; doi:10.3724/abbs.2023129)
Supplement: 23276Supplementary_materials [file 23276Supplementary_materials.pdf]

Figure S1

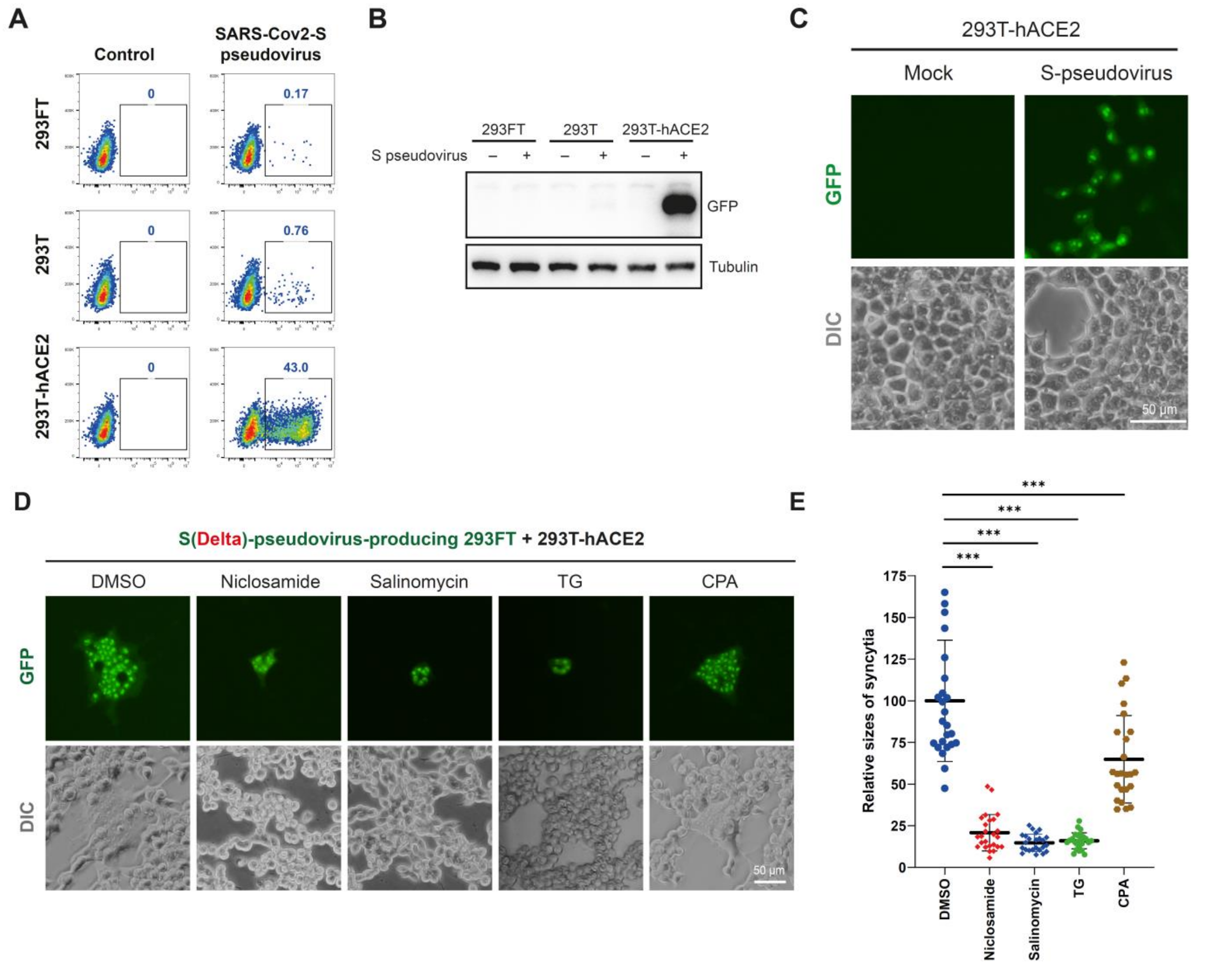

Supplementary Figure S1. 293FT generating S pseudovirus can induce cell fusion in hACE2-positive 293T cells (A,B) SARS-CoV-2 S pseudovirus can effectively infect 293T-hACE2, but not 293T or 293FT cells. 293T, 293FT or 293T-hACE2 cells were incubated with 1 mL DMEM containing S pseudovirion, and seeded in 12-well plates. 24 h later, culture media were changed to fresh DMEM. Cells were cultured for another 24 h. Infection efficiencies were analyzed by (A) flow cytometry or (B) anti-GFP western blot analysis. (C) S pseudovirus infection in 293T-hACE2 cells cannot induce syncytia formation. 293T-hACE2 cells were infected by S pseudovirus supernatant. After 48 h, images were taken under an Olympus IX73 microscope. Scale bar: 50 μm. (D,E) This pseudovirus-based method can be used to estimate the inhibitory effects of drugs on Delta S-mediated cell fusion. DMSO, niclosamide (1 μM), salinomycin (1 μM), thapsigargin (TG) (1 μM) or cyclopiazonic acid (CPA) (5 μM) was added to the mixture of 293T-hACE2 cells and 293FT cells producing Delta S pseudovirus. Images are shown in (D), and the quantitative analysis of the syncytia sizes is shown in (E). Scale bar: 50 μm. Data are presented as the mean ± SD, ****P*<0.001. (Student's *t*-test, *n*=20).

Supplementary Video S1. A pseudovirus-producing 293FT induces syncytia formation in 293T-hACE2 cells A single 293FT cell generating S pseudovirus was mixed with 293T-hACE2 cells. Cells were seeded in 29-mm dish and monitored with the Olympus SpinSR real-time live cell imaging system. Cell images were acquired every 5 min for total 15

h and then combined to generate a video.

Materials and Methods

Cell lines

Human embryonic kidney cell line 293T and 293FT, and Calu-3 cells were cultured in Dulbecco's modified Eagle medium (DMEM) containing 100 units/mL penicillin, 100 mg/mL streptomycin, 2 mM L-glutamine and 10% fetal bovine serum (FBS), at 37ºC with 5% CO_2. 239T cell line stably expressing human ACE2 (293T-hACE2) was constructed by hACE2 overexpression.

Plasmids and reagents

pSPAX2 was obtained from Addgene (Watertown, USA). pCDH-sfGFP was provided by Dr Qiang Deng from Fudan University (Shanghai, China). The pVAX1 vectors encoding several S variants (WT, D614G, Alpha, Beta, and Delta) were generously offered by Prof Dimitri Lavillette from Institut Pasteur of Shanghai (Shanghai, China). Omicron S gene was synthesized and cloned to pVAX1.

The anti-GFP (#RLI-09) mouse antibody was provided by Biolinkedin (Shanghai, China). Niclosamide (HY-B0497) and Salinomycin (HY-15597) were ordered from MedChemExpress, (Monmouth Junction, USA). Thapsigargin (SC0389-2mM) was obtained from Beyotime Biotechnology (Shanghai, China), and cyclopiazonic acid (T15027) was from TargetMol Chemicals Inc. (Boston, USA).

Production and transduction of SARS-CoV-2 S pseudovirions

Similar to a previous study [1], psPAX2, pCDH-sfGFP and an S-expressing plasmid (ratio: 4.5:3:3) were co-transfected into 293FT cells using Lipofectamine 2000 (Invitrogen, Carlsbad, USA). Forty-eight hours later, virus supernatants were collected and passed through 0.45-μm filters, and then stored at –80ºC. For pseudovirus transduction, 293T-hACE2 cells were digested into single cells by using trypsin-EDTA. 293T-hACE2 cells were incubated in 1 mL DMEM containing pseudovirus, and seeded in 12-well plates. After 24 h of incubation at 37ºC, culture media were changed to fresh DMEM. Cells were further cultured for 24 h, and digested with trypsin. Flow cytometric analysis was used to determine the percentages of GFP-positive cells.

Pseudovirus-based cell-cell fusion assay

To prepare virions-producing cells, psPAX2, pCDH-sfGFP and an S-expressing plasmid were co-transfected into 293FT cells at a ratio of 4.5:3:3 using Lipofectamine 2000 for 24 h. Pseudovirus-producing 293FT cells and 293T-hACE2 cells were digested into single cells by using trypsin, and cocultured at a ratio of more than 1:500. Cell mixture was then maintained in 37ºC for 12–24 h. Live cell imaging was conducted with Olympus IX73 microscope or Olympus SpinSR system (Olympus, Tokyo, Japan).

Confocal fluorescence microscopy

According to a previous confocal protocol [2], cells were fixed in 4% PFA at room temperature for 10–20 min, and washed with DPBS for three times. Cells were then stained with 10 μM DiI (red) for 30 min. After cells were washed with DPBS for three times, DAPI was utilized for nuclear staining. Confocal imaging was performed with the Olympus SpinSR imaging system.

Statistical analysis

All data are presented as the mean ± SD. GraphPad Prism was used for data analysis. Statistical significance was determined by Student's *t*-test. P values < 0.05 were considered to be statistically significant.

Reference

1. Ou X, Liu Y, Lei X, Li P, Mi D, Ren L, Guo L, Guo R, Chen T, Hu J: Characterization of spike glycoprotein of SARS-CoV-2 on virus entry and its immune cross-reactivity with SARS-CoV. *Nature communications* 2020, **11**(1):1-12.
2. Sheng X, You Q, Zhu H, Li Q, Gao H, Wang H, You C, Meng Q, Nie Y, Zhang X *et al*: Enterohemorrhagic E. coli effector NleL disrupts host NF-κB signaling by targeting multiple host proteins. *Journal of Molecular Cell Biology* 2020, **12**(4):318-321.
